# Dispensable role of Rac1 and Rac3 after cochlear hair cell specification

**DOI:** 10.1007/s00109-023-02317-4

**Published:** 2023-05-19

**Authors:** Takashi Nakamura, Hirofumi Sakaguchi, Hiroaki Mohri, Yuzuru Ninoyu, Akihiro Goto, Taro Yamaguchi, Yoshitaka Hishikawa, Michiyuki Matsuda, Naoaki Saito, Takehiko Ueyama

**Affiliations:** 1 Laboratory of Molecular Pharmacology, Biosignal Research Center, Kobe University, Kobe, 657-8501 Japan; 2 Department of Otolaryngology-Head and Neck Surgery, Kyoto Prefectural University of Medicine, Kyoto, 602-8566 Japan; 3 Graduate School of Biostudies, Kyoto University, Kyoto, 606-8315 Japan; 4 Laboratory of Pharmacology, Faculty of Pharmaceutical Sciences, Setsunan University, Hirakata, 573-0101 Japan; 5 Department of Anatomy, Histochemistry and Cell Biology, Faculty of Medicine, University of Miyazaki, Miyazaki, 889-1692 Japan

**Keywords:** Atoh1, Development, Hearing, Maintenance, Rho-family small GTPases

## Abstract

**Abstract:**

Rac small GTPases play important roles during embryonic development of the inner ear; however, little is known regarding their function in cochlear hair cells (HCs) after specification. Here, we revealed the localization and activation of Racs in cochlear HCs using GFP-tagged Rac plasmids and transgenic mice expressing a Rac1-fluorescence resonance energy transfer (FRET) biosensor. Furthermore, we employed *Rac1*-knockout (*Rac1*-KO, *Atoh1-Cre;Rac1*^*flox/flox*^) and *Rac1* and *Rac3* double KO (*Rac1/Rac3*-DKO, *Atoh1-Cre;Rac1*^*flox/flox*^*;Rac3*^*−/−*^) mice, under the control of the *Atoh1* promoter. However, both *Rac1*-KO and *Rac1/Rac3*-DKO mice exhibited normal cochlear HC morphology at 13 weeks of age and normal hearing function at 24 weeks of age. No hearing vulnerability was observed in young adult (6-week-old) *Rac1/Rac3*-DKO mice even after intense noise exposure. Consistent with prior reports, the results from *Atoh1-Cre;tdTomato* mice confirmed that the *Atoh1* promoter became functional only after embryonic day 14 when the sensory HC precursors exit the cell cycle. Taken together, these findings indicate that although Rac1 and Rac3 contribute to the early development of sensory epithelia in cochleae, as previously shown, they are dispensable for the maturation of cochlear HCs in the postmitotic state or for hearing maintenance following HC maturation.

**Key messages:**

- Mice with Rac1 and Rac3 deletion were generated after HC specification.
- Knockout mice exhibit normal cochlear hair cell morphology and hearing.
- Racs are dispensable for hair cells in the postmitotic state after specification.
- Racs are dispensable for hearing maintenance after HC maturation.

**Supplementary Information:**

The online version contains supplementary material available at 10.1007/s00109-023-02317-4.

## Introduction

The mammalian inner ear is a highly elaborate sensory organ specialized for hearing and balance perception; its developmental morphology is well understood in mice. The inner ear is generated from a small patch of thickened ectoderm; this otic placode gradually begins to invaginate from embryonic days 8–9 (E8–9) forming the otocyst at E9.5 [1]. By E10.5, the developing cochlear duct protrudes from the otocyst and begins to form a spiral by E12.5, gradually elongating between E12.5 and E17.5, together with the sensory primordium, to ultimately form approximately one and three-quarter turns [2]. During this period, the sensory primordium gives rise to the organ of Corti (OC) comprising mechanosensory hair cells (HCs) and non-sensory supporting cells (SCs) [3].

Atonal homolog 1 (ATOH1) is a basic helix-loop-helix transcription factor essential for HC differentiation and an early marker of HC differentiation [4]. In HCs, ATOH1 expression begins when sensory precursors exit the cell cycle [5–7], first appearing in the mid-basal region of the sensory epithelium around E14 and then spreading bidirectionally across the developing OC [7, 8]. This cochlear patterning process is completed around E18-P0 [5, 9, 10]. The resulting HCs exhibit orderly arranged stereocilia composed of high-density parallel actin filaments on the apical surface. ATOH1 expression is also required for HC maturation, including that of stereocilia, which underlie the mechanosensory function in hearing [6, 11, 12]. Specifically, stereocilia deflections modulate the positive ion current flowing into HCs to generate an intracellular receptor potential. However, even if HCs mature normally, they may subsequently be impaired by various factors, including aging, acoustic trauma, ototoxic drugs, and infection. Once HCs are lost, they are not usually replaced, resulting in sensorineural hearing loss (SNHL) [6].

Mammalian Rac, a member of the Rho-family of small GTPases, comprises three isoforms, Rac1, Rac2, and Rac3, which share high (88–93%) amino acid identity in mice and humans. Rac1 is ubiquitously expressed, whereas Rac2 and Rac3 are predominantly expressed in hematopoietic and neuronal cells, respectively [13, 14]. Rac1 and Rac3 function in cell polarity, cell–cell adhesion, cell migration, cell cycle progression, transcriptional activation, and cell death [14], which are essential for organ development and differentiation along with subsequent maintenance. In particular, we previously reported that Rac1 and Rac3, to a lesser extent, function in cerebellar granule neurons during cerebellar development [15]. In the inner ear, *Rac1*-knockout (KO) mice exhibit severely shortened cochleae and distinctly smaller vestibules and semicircular canals [16], whereas *Rac1* and *Rac3* double KO (DKO) mice show enhanced dysmorphic phenotypes in cochleae, vestibules, and semicircular canals [17], suggesting that Rac1 and Rac3 have partially redundant functions during inner ear development. Notably, these studies [16, 17] incorporated Cre recombinase driven by the *Foxg1* or *Pax2* promoter to delete Racs from the inner ears. Notably, Cre expression levels are reportedly higher in *Foxg1-Cre* than *Pax2-Cre* mice [18]. The *Foxg1* [19] and *Pax2* [20] promoters function in otic placodes at E8.5 prior to the expression of ATOH1 (~ E14). Additionally, these promoters are active in the telencephalon (*Foxg1*), brain stem, and throughout the OC epithelia, including HCs, SCs, and their precursors [21, 22]; consequently, conditional *Rac1*-KO and *Rac1/Rac3*-DKO mice under the control of Foxg1-Cre or Pax2-Cre die at birth [16, 17]. However, HCs continue to differentiate after birth to function as mechanosensory transmitters; for example, the precise number/row, length, width, and links of HC stereocilia are established by postnatal day 20 (P20) [23, 24]. Thus, the function of Racs after HC specification remains unclear.

In the current study, we generated conditional KO mice under the control of the *Atoh1* promoter: *Atoh1-Cre;Rac1*^*flox/flox*^ (*Rac1*-KO) and *Atoh1-Cre;Rac1*^*flox/flox*^*;Rac3*^*−/−*^ (*Rac1/Rac3*-DKO) mice to examine the role of Racs in cochlear HCs. Rac1/Rac3-DKO showed no effect on HC morphology and function.

## Materials and methods

### Animals

*Atoh1-Cre* TG mice [25], *Rac1*^*flox/flox*^ mice [26], and *Rac3*^*−/−*^ mice [27] were backcrossed to generate *Atoh1-Cre;Rac1*^*flox/flox*^ mice (*Atoh1-Cre*^*−/−*^*;Rac*^*flox/flox*^ as a control, *Atoh1-Cre*^+*/−*^*;Rac1*^*flox/flox*^ as *Rac1*-KO) and *Atoh1-Cre;Rac1*^*flox/flox*^*;Rac3*^*−/−*^ mice (*Atoh1-Cre*^+*/−*^*;Rac1*^*flox/flox*^*;Rac3*^*−/−*^ as *Rac1/Rac3*-DKO) [15]. *CAG-STOP*^*flox*^*-tdTomato* (Ai9) mice were purchased from the Jackson Laboratory (Bar Harbor, ME, USA) and backcrossed to generate *Atoh1-Cre;tdTomato* mice, which were used to examine the function of the *Atoh1* promoter in cochleae. The efficacy of *Atoh1-Cre* mice in cochleae has been previously reported [25, 28].

Offspring were genotyped via PCR using the following primer pairs: *Atoh1-Cre* (5′-GCATACCTGGAAAATGCTTC-3′ and 5′-CCAGTGAAACAGCATTGCTG-3′), *Rac1*^*flox*^ (5′-ATTTTCTAGATTCCACTTGTGAAC-3′ and 5′-ATCCCTACTTCCTTCCAACTC-3′), *Rac3*^*−*^ (5′-CATTTCTGTGGCGTCGCCAAC-3′ and 5′-TTGCTGGTGTCCAGACCAAT-3′), *Rac3*^+^ (5′-CATTTCTGTGGCGTCGCCAAC-3′ and 5′-CACGCGGCCGAGCTGTGGTG-3′), and *tdTomato* (5′-GGCATTAAAGCAGCGTATCC-3′ and 5′-CTGTTCCTGTACGGCATGG-3′).

Mice were housed under specific pathogen-free conditions using an individually ventilated cage system (Techniplast, Tokyo, Japan). Both male and female mice were included in analyses unless otherwise indicated (mice younger than 1 week were not differentiated based on sex). Age- and sex-matched siblings were used as controls.

### ISH, DNA microarray, and RT-PCR

In situ hybridization (ISH) was performed using the cochleae from P6 WT mice (CLEA Japan, Tokyo) as previously described [28]. Briefly, the following 45 bp DNA probes labeled at their 5′-end with digoxigenin-11-dUTP were used: *Rac1* (antisense [nucleotides 346–390 from ATG]: 5′-GAGCAGGCAGGTTTTACCAACAGCTCCGTCTCCCACCACCACACA-3′ and sense: 5′-TGTGTGGTGGTGGGAGACGGAGCTGTTGGTAAAACCTGCCTGCTC-3′), and *Rac3* (antisense [155–199]: 5′-CAGCAGGCACGTCTTCCCCACGGCACCATCGCCAACCACCACGCA-3′ and sense: 5′-TGCGTGGTGGTTGGCGATGGTGCCGTGGGGAAGACGTGCCTGCTG-3′). ISH signals were detected immunohistochemically; the staining intensities of the sense and antisense probes were compared using a digital image analyzer (WinRoof version 7.0, Mitani Corp., Tokyo, Japan).

DNA microarray analysis was performed as previously described [26]. Total RNA was extracted from the cochleae of five P6 WT mice using a NucleoSpin RNA kit (MACHEREY–NAGEL GmbH & Co. KG, Düren, Germany). Gene expression profiles were examined using the SurePrint G3 Mouse GE 8 × 60 K Microarray Kit (Agilent Technologies, Santa Clara, CA, USA).

RT-PCR was performed with 1 µg of total RNA obtained from 14 membranous cochleae and vestibules of 4-week-old WT mice using SuperScript III reverse transcriptase (Invitrogen, Carlsbad, CA, USA), as previously described [26]. The following primer pairs were used: 5′-GCAGACAGACGTGTTCTTAATTTGC-3′ and 5′-CAACAGCAGGCATTTTCTCTTCC-3′ for *Rac1* (predicted product size: 358 bp); 5′-GGAGGACTATGACCGCCTC-3′ and 5′-GCGCTTCTGCTGTCGTGTG-3′ for *Rac2* (379 bp); and 5′-CCCACACACACCCATCCTTC-3′ and 5′-CAGTGCACTTCTTGCCTGGC-3′ for *Rac3* (257 bp).

### Plasmids and transfection of organotypic cochlear explant cultures

*Rac1* and *Rac3* in the pEGFP(C1) vector (Takara Bio, Kusatsu, Japan; termed EGFP-Rac1 and EGFP-Rac3, respectively) have been previously described [13]. Organotypic OC explant cultures were prepared from WT P4 rats as previously described [28]. For transfection, a Helios Gene Gun (Bio-Rad Laboratories, Hercules, CA, USA) and Helios Gene Gun Diffusion Screen (165–2475) were used, which reduce tissue damage owing to the high concentration of gold particles in the center of the shot. Gold particles (1.0 μm diameter) were coated with the plasmids at a ratio of 2 μg plasmid to 1 mg gold particles and precipitated onto the inner wall of Tefzel tubing, which was cut into individual cartridges containing 1 μg of the plasmid. The next day (ex vivo day 1), the samples were bombarded with gold particles from one cartridge per culture using 110 psi helium pressure, as previously described [29]. The explants were fixed 24 h after transfection with 4% paraformaldehyde in 0.1 M phosphate buffer (pH 7.4), counterstained with Alexa568-conjugated phalloidin, and observed under an LSM700 confocal microscope (Carl Zeiss, Oberkochen, Germany).

### FRET imaging

OCs from P2 *Rac1-*fluorescence resonance energy transfer (FRET) biosensor TG mice [30] were dissected in Leibovitz’s L-15 medium (Invitrogen), attached to 3.5-mm Cell-Tak coated dishes (150 µg/µL; BD Biosciences) and maintained in Dulbecco’s modified Eagle medium/F-12 supplemented with 10% fetal bovine serum. FRET imaging under a two-photon excitation microscope was performed as previously described [28]. Samples were maintained in an incubation chamber (Tokai Hit, Nagoya, Japan) and imaged using a BX61WI/FV1000 upright microscope equipped with a × 60 water-immersion objective (LUMPlanFLN; Olympus, Tokyo, Japan) connected to a Mai Tai DeepSee HP Ti:sapphire laser (Spectra Physics, Mountain View, CA, USA). FRET/CFP images were acquired and analyzed using MetaMorph (Universal Imaging, West Chester, PA, USA) and Imaris software (Bitplane AG, Zürich, Switzerland) and represented using the intensity-modulated display mode, in which eight colors from red to blue are used to represent the FRET/CFP ratio.

### ABR measurement and noise exposure (NE)

Auditory brainstem responses (ABRs) were obtained under anesthetization with a mixture of medetomidine, midazolam, and butorphanol (intraperitoneal injection, 0.3, 4.0, and 5.0 mg/kg, respectively) on a heating pad, as previously described [31]. Briefly, ABR waveforms using sound stimuli of clicks or tone bursts at 8, 16, 24, or 32 kHz were recorded and averaged. ABR waveforms were recorded using elicitation sound that ranged from 100 to 5 dB SPL, and the thresholds (dB SPL) were defined by decreasing the sound intensity by 5 dB intervals until the lowest sound intensity level was reached, resulting in a recognizable ABR wave pattern (primarily judged by recognition of wave III).

NE experiments were performed as previously described [31]. Briefly, 6-week-old control and *Rac1/Rac3*-DKO mice were anesthetized and exposed to 110 dB SPL octave-band noise centered at 8 kHz for 1 h inside a sound chamber. These NE conditions cause a permanent threshold shift in WT mice [31]. ABR thresholds (dB SPL, at 4, 12, and 20 kHz) were measured immediately before NE and were measured sequentially after NE on day 0 and days 2, 7, and 14. NE-induced hearing deterioration was evaluated using the ABR threshold shift, calculated based on differences in the ABR threshold before and after NE.

### Immunohistochemistry

To examine cochlear whole mounts, surface preparations, and cryostat sections, tissues were fixed with 4% paraformaldehyde in 0.1 M phosphate buffer, as previously described [31]. Samples for surface preparations and cryostat sections were decalcified in 0.12 M ethylenediaminetetraacetic acid for 1 week at 4 °C or for 2 days at 23 °C. After permeabilization with phosphate-buffered saline containing 0.3% Triton X-100, the samples were incubated with Alexa Fluor 488-labeled phalloidin (Invitrogen) with/without DAPI for 1 h at 23 °C. The stained tissues were mounted in Prolong anti-fade (Invitrogen) with a coverslip and observed using an LSM700 confocal microscope.

#### SEM

Scanning electron microscopy (SEM) analysis was performed as previously described [32]. Freshly dissected cochleae of 13-week-old WT, *Rac1*-KO, and *Rac1/Rac3-DKO* mice were fixed with 2% paraformaldehyde and 2.5% glutaraldehyde in 0.1 M phosphate buffer for 2 h, followed by post-fixation with 1% osmium tetroxide in H_2_O for 1 h at 23 °C. Tissues were dehydrated using a graded ethanol series, followed by tert-butyl alcohol, and dried in a vacuum freeze dryer (VFD-30; Ulvac Inc., Tokyo, Japan). Dried tissues, mounted on stages, were sputter coated with gold in an Ion Sputter MC1000 (Hitachi High-Tech Corp., Tokyo, Japan) and observed using a TM3030Plus scanning electron microscope (Hitachi High-Tech).

### Statistical analysis

Blinded data analysis was performed by two otologists or scientists. Statistical analyses were performed with Prism 7.0 software (GraphPad Software Inc., La Jolla, CA, USA) using two-way analysis of variance (ANOVA) followed by Tukey’s post-hoc test. Statistical significance was set at *P* < 0.05.

## Results

### Expression of Rac1 and Rac3 in cochlear HCs

**Fig. 1:**
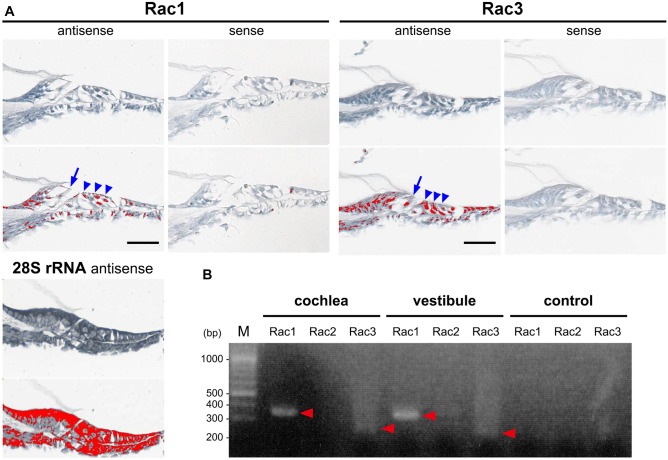
Expression of *Rac1* and *Rac3* in cochlear hair cells (HCs). **A** In situ hybridization (upper panels) of *Rac1* and *Rac3* mRNA expressions in cochlear inner HCs (IHCs; arrows) and outer HCs (OHCs; arrowheads) in P6 wild-type (WT) mice. The lower panels show the relative *Rac1* and *Rac3* mRNA signal levels as determined using an image analyzer (red was assigned as positive). A 28S rRNA antisense oligo-DNA probe was used as a positive control. The data shown is representative of at least three experiments. Scale bars: 50 µm. **B** RT-PCR was performed using total RNA from 4-week-old WT cochleae and vestibules and specific primer pairs (*Rac1*, *Rac2*, and *Rac3* predicted product sizes of 358, 379, and 257 bp, respectively). Arrowheads indicate the specific bands detected. PCR without cDNA served as a negative control. The data shown is representative of at least three experiments

We confirmed the expression of *Rac1* and *Rac3* mRNA in cochlear inner HCs (IHCs) and outer HCs (OHCs) using ISH in P6 WT mice (Fig. 1a). To evaluate *Rac1* and *Rac3* expression in cochleae, DNA microarray analysis was performed on P6 WT mice. *Rac1* mRNA expression was predominant (*Rac1* expression [28529.7] was 7.6-fold higher than that of *Rac3* [3752.0]). Furthermore, RT-PCR analysis revealed clear *Rac1* and faint *Rac3* bands from the cochleae and vestibules of WT mice at 4 weeks of age (Fig. 1b).

### Localization and activation of Rac1 and Rac3 in cochlear HCs

**Fig. 2:**
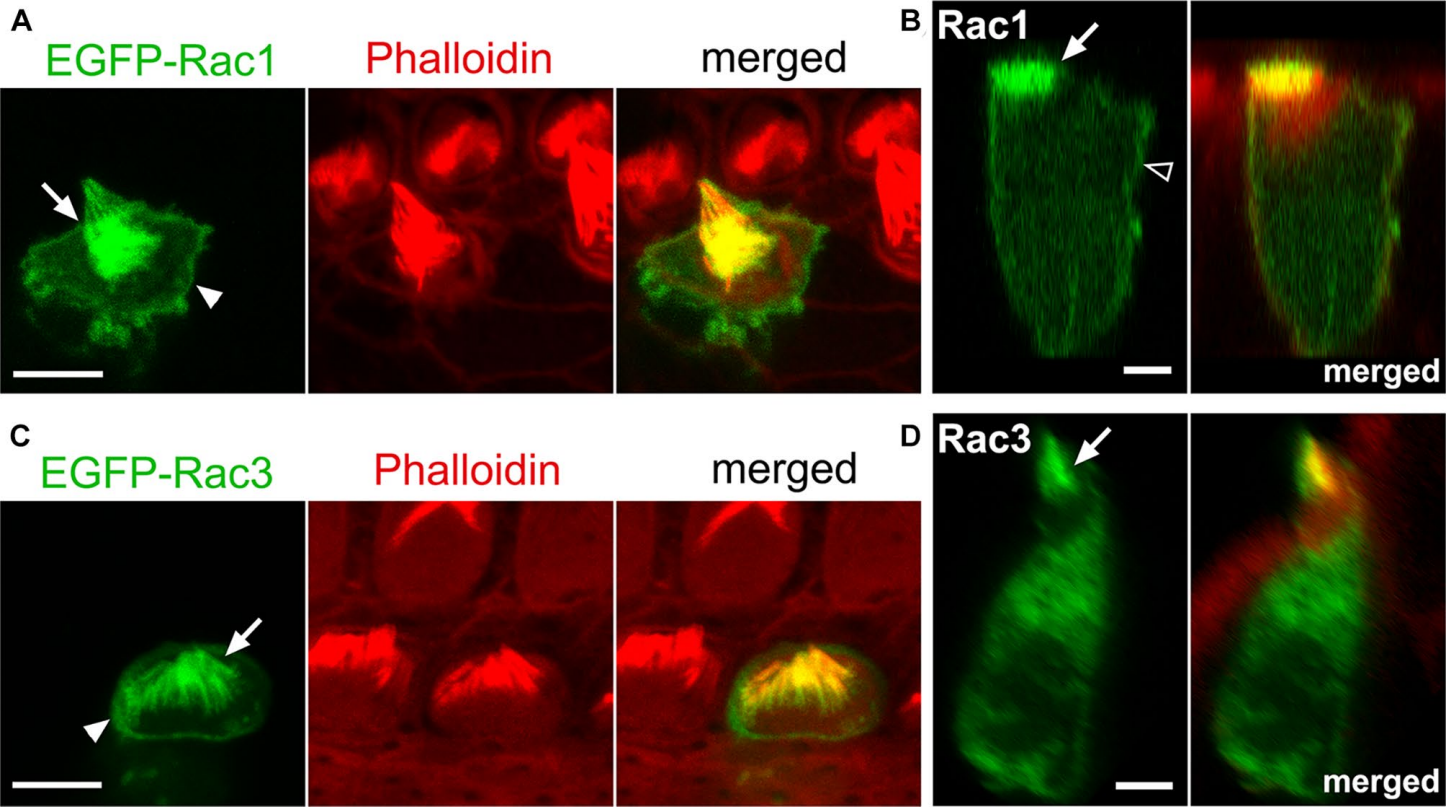
Rac1 and Rac3 localization at stereocilia, apical cell junctions, and lateral membranes in cochlear HCs. Dissected organs of Corti (OCs) from P4 WT rats were cultured for 16 h and transfected with the indicated EGFP-tagged Rac plasmids using a gene gun. **A**, **C** EGFP-tagged Rac1 and Rac3 (green) are localized at stereocilia (arrows) and apical cell junctions in outer HCs (arrowheads). **B**, **D** Reconstructed lateral view images of the same OHCs shown in A and C. EGFP-tagged Rac1 and Rac3 localized at stereocilia (arrows). EGFP-tagged Rac1, but not Rac3, localized in the lateral membranes of the OHC (open arrowhead). *n* ≥ 4 in each (Rac1 and Rac3) group. Scale bars: 5 µm

Next, we examined the localization and activation of Rac1 and Rac3 in cochlear HCs. Using a gene gun, EGFP-tagged Rac1 or Rac3 was transfected into organotypic cochlear explants obtained from P4 WT rats. Intense EGFP-Rac1 and EGFP-Rac3 fluorescence was observed in the stereocilia of cochlear HCs (Fig. 2a–d). Additionally, both EGFP-Rac1 and EGFP-Rac3 were localized at the apical cell junctions of cochlear HCs (Fig. 2a, c). EGFP-Rac1, not EGFP-Rac3, fluorescence was localized to the lateral membranes of cochlear HCs (Fig. 2b, d). These results were consistent with our previous report [13] that Rac1 accumulates more strongly at the plasma membrane than Rac3.

**Fig. 3:**
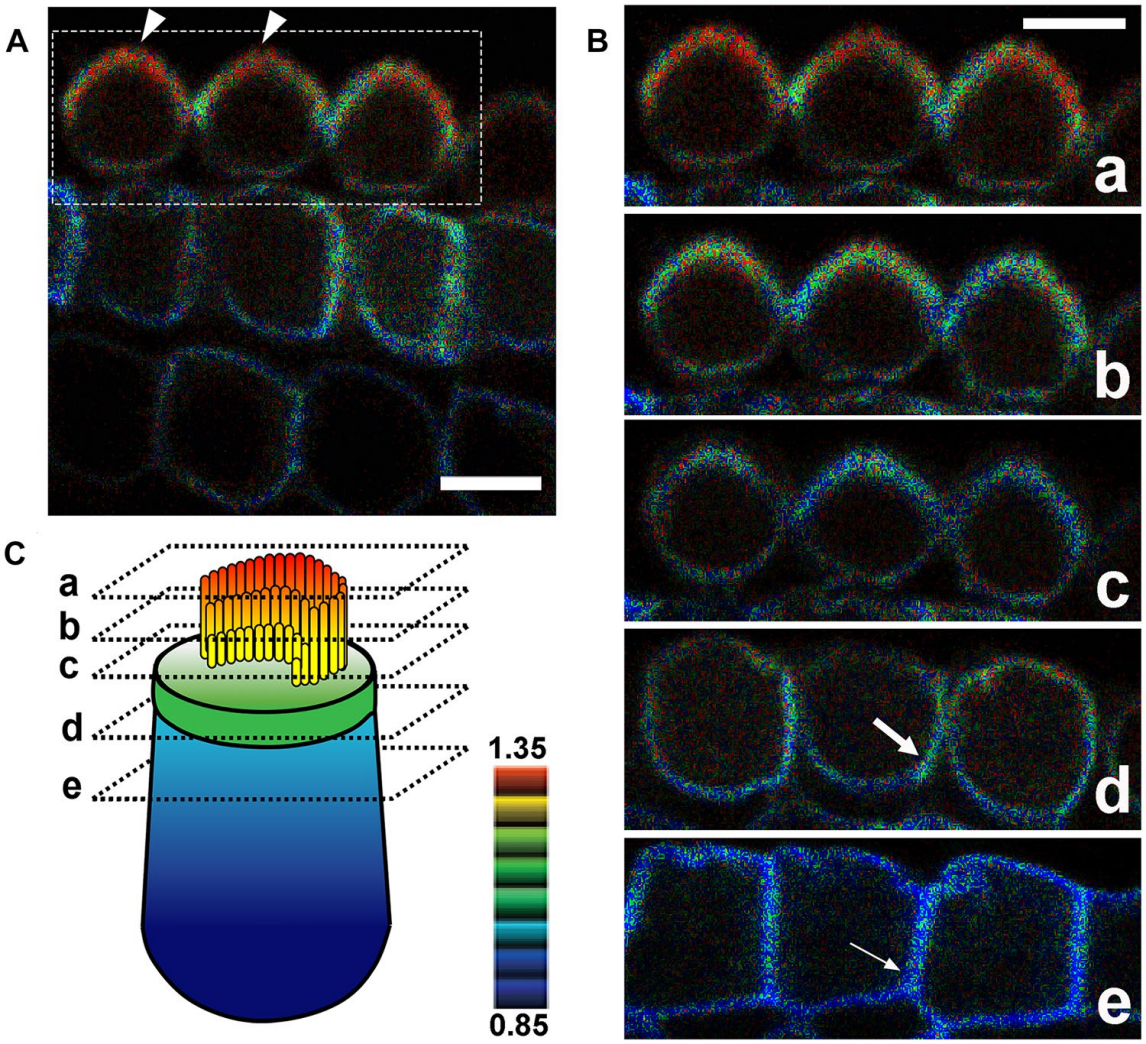
Rac1 activity in cochlear OHCs. Dissected OCs from P2 *Rac1-FRET* TG mice were observed under a two-photon excitation microscope. **A** In OHCs, the FRET/CFP ratio is the highest in stereocilia (arrowheads). A movie is available in Online Resource 3. **B** Magnified composite images of the region indicated by the dotted box in (**A**) showing the FRET/CFP ratios of a series of three OHCs obtained in serial sections from the base to the top of the OHCs, which were extracted from Online Resource 3. In all three OHCs, the FRET/CFP ratio is highest in stereocilia, and higher in the apicolateral membranes (large arrow) than in the basolateral membranes (small arrow). **C** Schematic drawing showing the levels of the composite images obtained from the OHCs shown in (**B**), along with the FRET/CFP ratio shown by a color gradient. *n* = 4. Scale bars: 10 µm

To ascertain whether Rac1 is activated/functions in stereocilia, apical cell junctions, and lateral membranes of cochlear HCs, we used transgenic mice (TG mice) expressing a Rac1 FRET biosensor [30]. Utilizing organotypic cochlear explants obtained from P2 *Rac1-FRET* TG mice, FRET images were obtained using a two-photon excitation microscope as previously described [28]. The FRET:CFP ratio (FRET/CFP) was most intense in stereocilia (Fig. 3a–c). In contrast, the FRET/CFP intensity at the lateral membranes was intermediate to low with an apical-to-basal gradient, and highest at apical cell junctions (Fig. 3b, c).

### Normal HC morphology and hearing in Rac1-KO and Rac1/Rac3-DKO mice

**Fig. 4:**
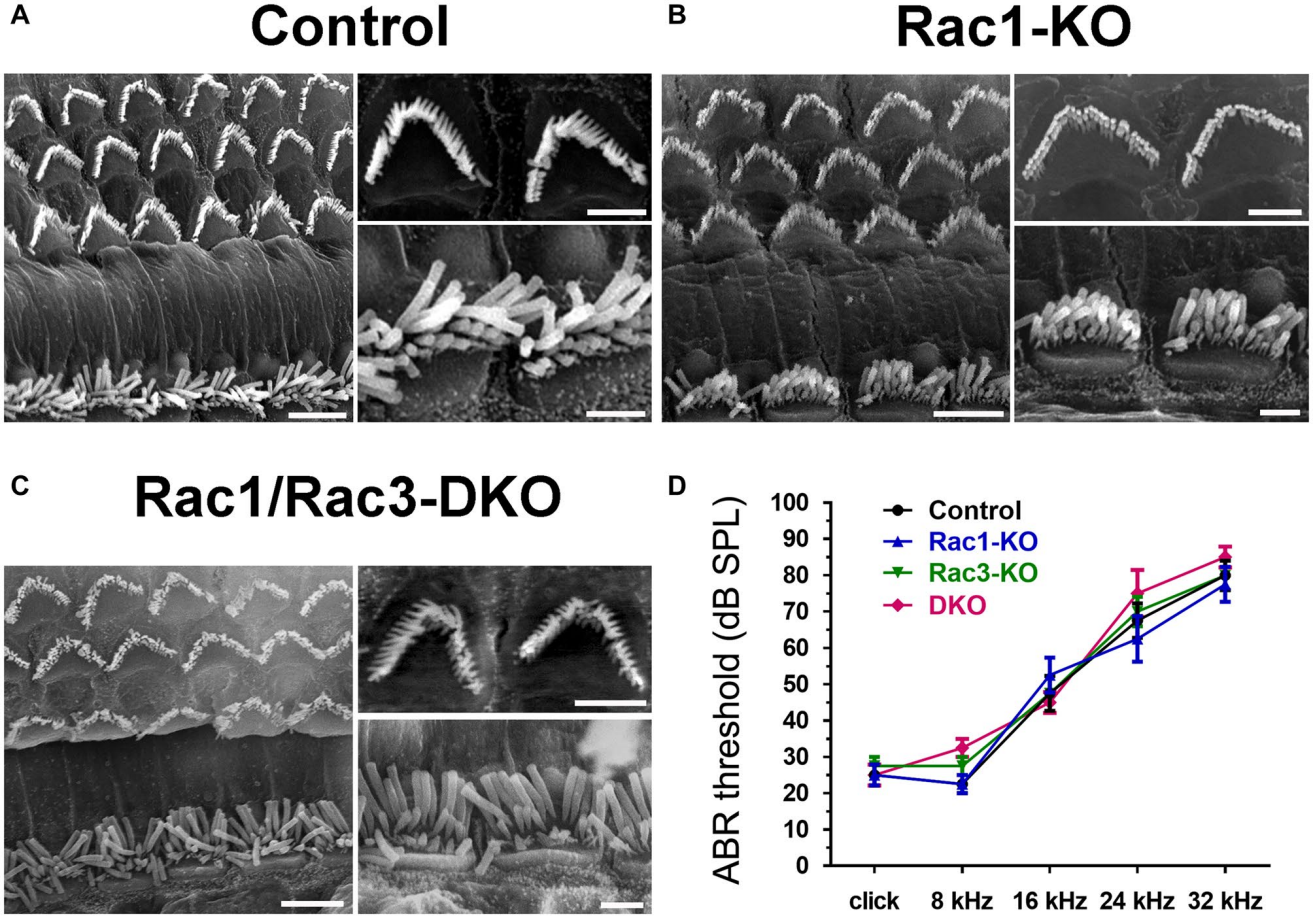
Normal HC morphology, no HC loss, and normal hearing in *Rac1*-KO and *Rac1/Rac3*-DKO mice. **A–C** OCs were prepared from 13-week-old control (**A**), *Rac1*-KO (**B**), and *Rac1/Rac3*-DKO mice (**C**), and images were obtained using a scanning electron microscope. The right panels of each figure are magnified images of OHCs (upper) and IHCs (lower). No significant differences were observed between groups (*n* = 4 in each group). Scale bars: 5 μm (large panels in **A**–**C**) or 2 μm (small panels in **A–C**). Additional magnified images of IHC stereocilia are provided in Online Resource 1. **D** ABR threshold was examined in 24-week-old control, *Rac1*-KO, *Rac3*-KO, and *Rac1/Rac3*-DKO mice. No significant differences were observed between groups, by two-way ANOVA with Tukey’s post-hoc test. *n* = 4 in each group

SEM of the middle turns of the cochleae at 13 weeks of age was examined to assess HC morphology in *Rac1*-KO and *Rac1/Rac3*-DKO mice. No difference was observed regarding HC loss between the control, *Rac1*-KO, and *Rac1/Rac3*-DKO mice (Fig. 4a–c, Online Resource 1). Additionally, normal arrangement and morphology of stereociliary bundles were observed in both IHCs and OHCs of the KO mice; these cells also exhibited normal planar cell polarity (PCP; Fig. 4a–c).

Additionally, the hearing function of *Rac1*-KO, *Rac3*-KO, and *Rac1/Rac3*-DKO mice was assessed at 24 weeks old using ABR. No significant difference was observed between the control (*Atoh1*^*−/−*^*;Rac1*^*flox/flox*^), *Rac1*-KO, *Rac3*-KO, and *Rac1/Rac3*-DKO mice (Fig. 4d).

### Hearing vulnerability is not detected in Rac1/Rac3-DKO mice after intense noise

**Fig. 5:**
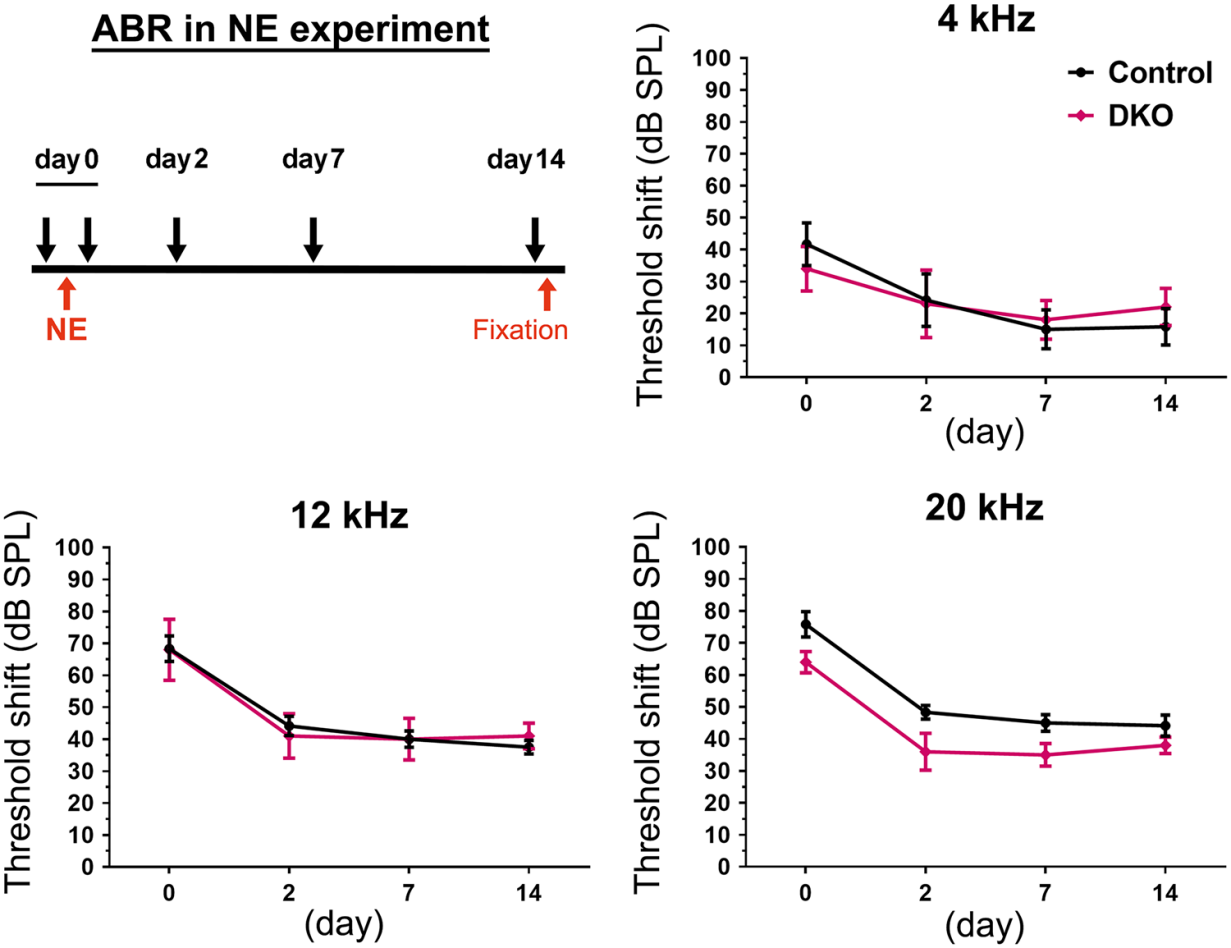
No hearing vulnerability following noise exposure (NE) in *Rac1/Rac3*-DKO mice. Six-week-old control (*n* = 6) and *Rac1/Rac3*-DKO (*n* = 5) mice were exposed to intense noise at 110 dB for 1 h. ABR thresholds at 4, 12, and 20 kHz were measured immediately before NE and sequentially after NE on days 0, 2, 7, and 14, as shown in the experimental scheme. Hearing deterioration was shown by the ABR threshold shift, calculated by the differences in ABR threshold before and after NE. No significant differences were observed between control and *Rac1/Rac3*-DKO mice, by two-way ANOVA with Tukey’s post-hoc test

To examine the vulnerability of hearing function in *Rac1/Rac3*-DKO mice, we exposed 6-week-old mice to NE with an intensity of 110 dB for 1 h. No significant difference in ABR threshold shifts at 4, 12, or 20 kHz was observed on days 0, 2, 7, and 14 following NE in *Rac1/Rac3*-DKO mice compared to control mice (Fig. 5).

### Evaluation of Atoh1 promoter function using Atoh1-Cre;tdTomato mice

**Fig. 6:**
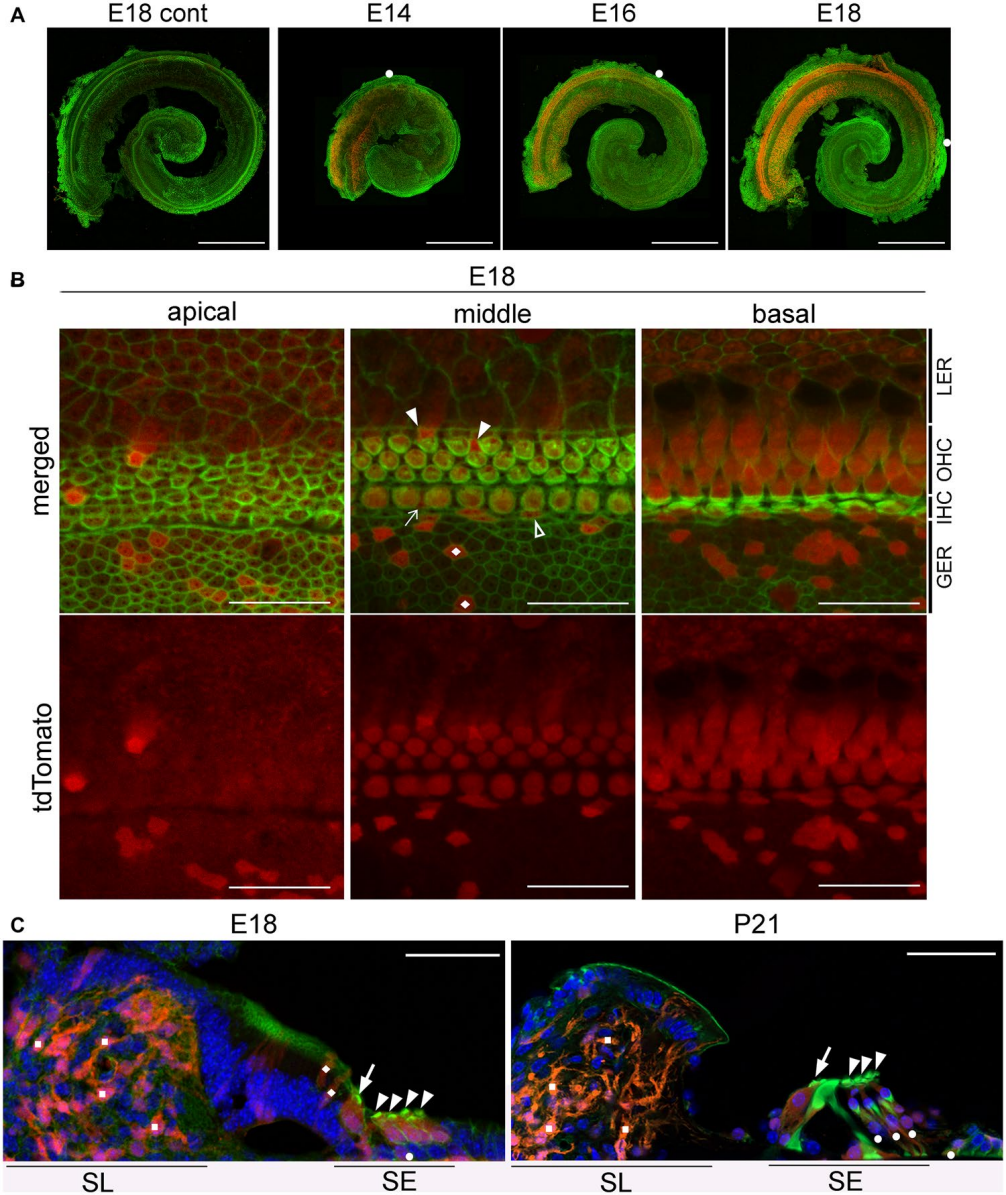
Time course of tdTomato expression in *Atoh1-Cre* TG mice. **A** Whole mount cochlea from E14, E16, and E18 *Atoh1-Cre;tdTomato* mice exhibit gradient expansion of tdTomato/Atoh1 expression in a basal-to-apical fashion. White circles indicate the apical tips of tdTomato-positive cells. The cochleae were stained with Alexa488-conjugated phalloidin (green). Scale bars: 500 μm. **B** Detailed localization of tdTomato-positive cells was ascertained using surface preparation of the OCs obtained from E18 *Atoh1-Cre;tdTomato* mice, stained with Alexa488-conjugated phalloidin (green). Most HCs, some supporting cells (SCs) in the sensory epithelium (Deiters’ cells: arrowheads; inner phalangeal cells: arrow; inner border cell: open arrowhead), and cells in the greater epithelial ridge (rhombi) are tdTomato-positive. LER, lesser epithelial ridge; GER, greater epithelial ridge. Scale bars: 20 μm. **C** In modiolar cryostat sections from E18 and P21 *Atoh1-Cre;tdTomato* mice, tdTomato-positive cells are located in the sensory epithelia (SE) as well as in the spiral limbus (SL). Based on location and morphology, tdTomato-positive cells are composed of IHCs (arrows), OHCs (arrowheads), some SCs (circles), cells in the GER (rhombi), and fibroblasts in the SL (squares). Scale bars: 50 μm. Images were obtained using a fluorescence microscope (**A**) and confocal microscope (**B**, **C**). *n* ≥ 3 per group in each experiment (**A**–**C**)

To clarify the discrepancy between the cochlear phenotypes of *Rac1*-KO and *Rac1/Rac3*-DKO mice obtained herein using the *Atoh1-Cre* driver and those reported previously using *Pax2-Cre* or *Foxg1-Cre* [16, 17], we evaluated the timing and cell-type specificity of *Atoh1* promoter activity using *Atoh1-Cre;tdTomato* mice. tdTomato-positive cells were first observed at E14 at the basal turn of the cochlea with progression to the apex (Fig. 6a). At E18, most IHCs and OHCs were positive for tdTomato fluorescence. Additionally, some SCs and cells in the greater epithelial ridge, which is a transient structure in the developmental process of the inner sulcus of the OC and possess cells with the ability to transdifferentiate into IHCs [33], were positive for tdTomato fluorescence (Fig. 6b). To further confirm the identity of tdTomato-positive cells in the OC, we evaluated cryostat sections of *Atoh1-Cre;tdTomato* cochleae and performed X-gal staining and Cre immunostaining to detect Cre expressing cells in *Atoh1-Cre;LacZ* mice. The surface preparation of *Atoh1-Cre*^+*/−*^*;LacZ* cochleae exhibited X-gal and Cre staining in OHCs and IHCs (Online Resource 2). The cryostat sections of *Atoh1-Cre*^+*/−*^*;tdTomato* cochleae showed tdTomato fluorescence in HCs and SCs, as well as in cells in the greater epithelial ridge (Fig. 6c). These data are consistent with previous reports in which *Atoh1* functions in the OC following differentiation of precursor cells into HCs and SCs [6]. Additionally, we observed tdTomato-positive cells in the spiral limbus (Fig. 6c). These cells are reportedly composed primarily of fibrocytes based on their morphology [34].

## Discussion

Herein, no morphological or hearing phenotypes were observed in *Atoh1-Cre-*driven *Rac1*-KO and *Rac1/Rac3*-DKO mice. Rac1 suppression at the apical membrane is considered essential for the maintenance of the renal cyst structure, as during the acquisition of cell polarity in Madin–Darby canine kidney (MDCK) cells, Rac1 activity is reportedly homogenous across the plasma membrane in early cystogenesis stages, however, is higher at the lateral membrane than the apical plasma membrane in later stages [35]. Additionally, an apicobasal gradient of Rac activity is required for the correct formation and positioning of protrusions in epithelial cells [36]. In comparison, in cochlear OHCs, we found that Rac is localized at the stereocilia, apical cell junctions, and lateral membranes, and that Rac1 activity, evaluated using OCs from *Rac1-FRET* TG mice, is highest at stereocilia and relatively higher at the apical than basal sides of the lateral membranes. This discrepancy could be explained by the peculiarity of cochlear HCs, which require PCP of HCs and SCs as well as cell-intrinsic planar polarity (apical–basal polarity) in individual HCs for proper development and maturation of the OC [37]. During stereocilia development and maturity, the establishment of cell-intrinsic planar polarity begins around E15, after HC specification [37], and is completed by ~ P20 together with the lengthening and widening of the stereocilia [24]. In the present study, Rac1 activity was examined using organotypic explants of cochleae at P2, when PCP had already been established in the OC, as well as in stereocilia during their development in HCs. Apical cell junctions are important for the development and maintenance of stereocilia [28, 38], which might account for why Rac1 activity was higher in stereocilia and apicolateral membranes than basolateral membranes. Importantly, the models employed in the current study (Rac-plasmid overexpression and TG mice for *Rac1*-FRET biosensor) may cause artificial effects on Rac localization and activity.

Using *Cdc42*-KO mice under the control of the *Atoh1* promoter (*Atoh1-Cre;Cdc42*^*flox/flox*^), we previously reported that Cdc42, a Rho-family small GTPase, plays essential roles in the maintenance of cochlear HCs [28]. After normal morphological maturation of cochlear HCs, *Cdc42*-KO mice exhibit progressive SNHL and HC loss, accompanied by various stereociliary abnormalities (e.g., scattered, short, long, and fused stereocilia). Subsequently, among patients with a heterozygous missense point mutation in CDC42, those with p.I21T, p.Y64C, p.R66G, or p.R68Q mutations reportedly manifest SNHL [39, 40]. Rho-family small GTPases have highly conserved switch I (residues 25–39) and switch II (residues 57–75) regions that participate in interactions with various guanine nucleotide exchange factors and GTPase-activating proteins [41]. Among the 13 reported CDC42 mutations [42], the activity of the p.I21T mutant protein remains unclear [39], whereas the p.Y64C, p.R66G, and p.R68Q mutations located in the switch II region are assumed to be constitutively active (dominant-active) mutations. Together, these results suggest that both activating and null mutations (represented by *Cdc42*-KO mice) cause SNHL, whereas recessive CDC42 mutations are likely embryonic lethal.

Seven patients with single substitution mutations in RAC1 have been reported (p.C18Y; p.N39S; p.V51M; p.V51L; p.C157Y, and p.Y64D and p.P73L in the switch II region) [43]. The p.C18Y and p.N39S, and p.Y64D mutations are assumed to be dominant-negative and dominant-active mutations, respectively [43]. Although patients carrying these mutations present with various central nervous system anomalies, including hypoplasia of the medial cerebellum and corpus callosum, consistent with the phenotypes of neuron-specific *Rac1*-KO mice [15, 44], only one patient with a dominant-active p.Y64D mutation presented with SNHL [43]. Additionally, although *Rac3*-KO mice show normal microscopic development of the brain [27], patients with RAC3 mutations (p.P29L and p.P34R in the switch I region and p.Q61L and p.E62K in the switch II region) reportedly exhibit severe intellectual disability and brain malformations [45, 46]. However, hearing function was not assessed. Moreover, patients with dominant-active, dominant-negative, or biallelic-null mutations in the hematopoietic cell-specific RAC2 isoform do not manifest SNHL [47, 48]. Conversely, although *Rac1*-KO and *Rac1/Rac3*-DKO mice under the control of the *Foxg1* or *Pax2* promoter died at birth, they exhibited severe defects in cochlear morphogenesis at E18.5, including short cochlea, reduced number of HCs, abnormal PCP, abnormal stereocilia, and mispositioning or absence of kinocilia [16, 17]. These phenotypes were enhanced in *Rac1/Rac3*-DKO mice, mediated by impaired cell adhesion, proliferation, and movement as well as increased cell death [17], suggesting that Racs (Rac1 and Rac3) exert their influence prior to the use of HCs. Together, these results suggest that both dominant-active and loss-of-function mutations of RAC1 cause morphological and functional anomalies in cochleae, whereas *Rac1*-KO and *RAC1* recessive mutations are likely embryonic lethal in mice and humans, respectively. Moreover, RAC1, which is the predominant RAC isoform during cochlear development, has a less substantial role in establishing/maintaining HC morphology and function than CDC42.

Although ATOH1 is also expressed in SCs, it is primarily expressed in HCs [21, 25, 49]. In our *Atoh1-Cre*-driven *Rac1*/*Rac3*-DKO mice, Racs were deleted from HCs but not from a large population of SCs after exiting the cell cycle (around E14), resulting in no hearing phenotypes even after NE in the permanent threshold shift model. However, given a temporary threshold shift following NE has been reported in association with a stereocilia anomaly [50], differences between the control and *Rac1/Rac3*-DKO mice might occur in a temporary threshold shift model. In sharp contrast, DKO of *Rac1* and *Rac3* in *Pax2-Cre* or *Foxg1-Cre* mice, in which Racs are deleted from HC and SC precursor cells in cochleae beginning at E8.5 [18], prior to HC specification/differentiation [16], resulted in various anomalies in cochleae and HCs. Hence, Racs are not likely essential during HC maturation or maintenance but rather contribute to the early development of cochlear sensory epithelia (before HC specification), including during cochlear growth and PCP establishment.

In summary, we demonstrated that Racs are dispensable in cochleae following HC specification. These findings are in sharp contrast to the important role that Cdc42 plays in the maintenance of HCs after HC specification. Additionally, the current study suggests that Racs may not affect Cdc42 in cochlear HCs. Further studies are required to evaluate the roles of other Rho-family small GTPases, which consist of 21 members [14] in hearing and cochlear development. These studies provide novel insights regarding the underlying mechanisms that can inform the development of therapeutics for SNHL with unknown etiology.

## Supplementary Information

Below is the link to the electronic supplementary material.

Supplementary file1 (TIF 22612 KB)

Supplementary file2 (TIF 14204 KB)

Supplementary file3 (MP4 4869 KB)

## Data Availability

All relevant data are within the manuscript and its supplementary information.
