# Supplementary figures and images for: Dispensable role of Rac1 and Rac3 after cochlear hair cell specification
Source: J Mol Med (Berl). 2023 May 19;101(7):843–54. doi: 10.1007/s00109-023-02317-4 (PMC10300165; doi:10.1007/s00109-023-02317-4)

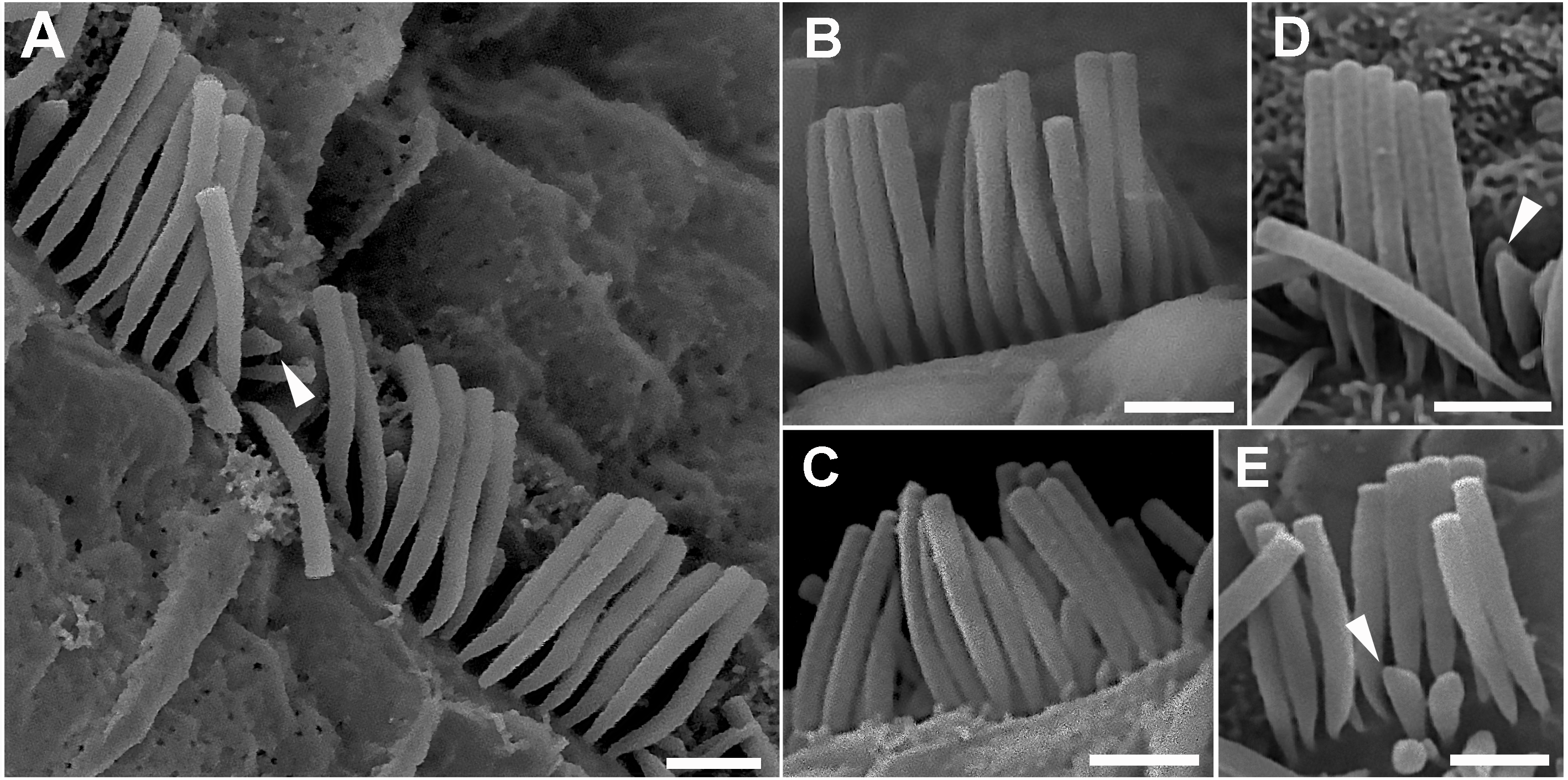

Supplement: Supplementary file 1 — Supplementary file1 (TIF 22612 KB) [file 109_2023_2317_MOESM1_ESM.tif]
